# Supplementary material for: FastqPuri: high-performance preprocessing of RNA-seq data
Source: BMC Bioinformatics. 2019 May 3;20:226. doi: 10.1186/s12859-019-2799-0 (PMC6500068; doi:10.1186/s12859-019-2799-0)
Supplement: Supplementary file 2 — Archive of FastqPuri. Archive containing all files needed to install and run FastqPuri v1.0.6. Date stamp March 22, 2019. (GZ 47,819 kb) [file 12859_2019_2799_MOESM2_ESM.gz › FastqPuri-1.0.6/html/md_README_makeTree.html]

My Project: makeTree user manual


My Project

makeTree user manual

Reads a `fasta` file, creates a tree structure of a predefined depth `DEPTH` and saves it to a file.

**NOTE**: computing a tree from a fasta file is very memory intensive. The program will not compute a tree if the length of the sequences in the fasta file exceeds 10 MB.

## Running the program

Usage `C` executable (in folder `bin`):

Usage: ./makeTree -f|--fasta <FASTA\_INPUT> -l|--depth <DEPTH>

-o, --output <OUTPUT\_FILE> Reads a \*fa file, constructs a tree of

depth DEPTH and saves it compressed in OUTPUT\_FILE.

Options:

-v, --version Prints package version.

-h, --help Prints help dialog.

-f, --fasta Input folder containing \*bin data (output from Qreport). Mandatory option.

-l, --depth depth of the tree structure

-o, --output Output file. If the extension is not \*gz, it is added. Mandatory option.

## Output description

Compressed file containing the tree structure. For further details, read the `Doxygen` documentation of the file `tree.c`, function `save_tree`.

## Example

TODOTODOTODOTODOTODO (It was tested somewhere!!)

## Contributors

Paula Pérez Rubio

## License

GPL v3 (see LICENSE.txt)


---

Generated by  

 1.8.14
